# 1-{[4-(4-{[(2-Oxidonaphthalen-1-yl)methyl­idene]aza­nium­yl}phen­oxy)phen­yl]iminiumylmeth­yl}naphthalen-2-olate

**DOI:** 10.1107/S1600536813007307

**Published:** 2013-03-23

**Authors:** Djahida Haffar, Djamel Daoud, Tahar Douadi, Leila Bouzidi, Salah Chafaa

**Affiliations:** a Laboratoire d’Électrochimie des Matériaux Moléculaires et Complexes (LEMMC), Département de Génie des Procèdes, Faculté de Technologie, Université FERHAT ABBAS – SETIF, 19000, Algeria

## Abstract

The title Schiff base compound, C_34_H_24_N_2_O_3_, was prepared by a condensation reaction of bifunctional aromatic diamine (4,4′-diamino­diphenyl ether) with hy­droxy­naphtaldehyde. The asymmetric unit contains two independent mol­ecules with similar conformations. The compound contains a central oxygen bridge and two functionalized [(*E*)-(phenyl­iminio)meth­yl]naphthalen-2-olate units. The dihedral angles between the benzene rings linking to the central O atom are 74.64 (19) and 69.85 (18)° in the two independent mol­ecules. Intra­molecular O—H⋯O hydrogen bonding occurs between the protonated imino N atoms and deprotonated hy­droxy O atoms in both mol­ecules. In the crystal, weak C—H⋯O hydrogen bonds are observed.

## Related literature

For biological and pharmacological activities of Schiff base compounds and their derivatives, see: Khandar *et al.* (2005 ▶); Chen *et al.* (2006 ▶); Kidwai *et al.* (2000 ▶); de Souza *et al.* (2005 ▶). For their application in water treatments, see: Izatt *et al.* (1995 ▶); Kalcher *et al.* (1995 ▶); Gilmartin & Hart (1995 ▶) and as corrosion inhibitors, see: Ahamad *et al.* (2010 ▶); Negm *et al.* (2010 ▶); Zhenlan *et al.*, (2002 ▶). For crystallographic studies of related compounds, see: Girija *et al.* (2004 ▶); Djamel *et al.* (2011 ▶); Gowda *et al.* (2007 ▶)*.* For the synthesis, see: Issaadi *et al.* (2005 ▶); Ghames *et al.* (2006 ▶).
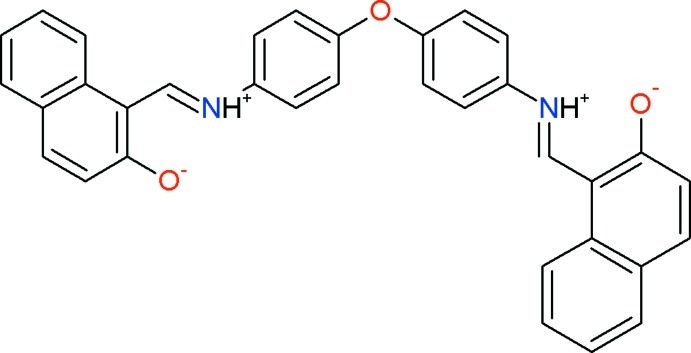


## Experimental

### 

#### Crystal data

C_34_H_24_N_2_O_3_
*M* *_r_* = 508.55
Triclinic, 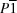

*a* = 5.292 (1) Å
*b* = 20.203 (1) Å
*c* = 23.863 (1) Å
α = 87.853 (10)°
β = 86.457 (10)°
γ = 85.26 (1)°
*V* = 2536.4 (5) Å^3^
*Z* = 4
Mo *K*α radiation
μ = 0.09 mm^−1^
*T* = 293 K
0.5 × 0.1 × 0.1 mm

#### Data collection

Nonius KappaCCD diffractometer
15547 measured reflections
9159 independent reflections
4705 reflections with *I* > 2σ(*I*)
*R*_int_ = 0.053

#### Refinement

*R*[*F* ^2^ > 2σ(*F* ^2^)] = 0.072
*wR*(*F* ^2^) = 0.218
*S* = 1.02
9159 reflections
706 parameters
H-atom parameters constrained
Δρ_max_ = 0.27 e Å^−3^
Δρ_min_ = −0.24 e Å^−3^

### 

Data collection: *COLLECT* (Nonius, 1999) ▶; cell refinement: *SCALEPACK* (Otwinowski & Minor, 1997 ▶); data reduction: *DENZO* (Otwinowski & Minor, 1997 ▶) and *SCALEPACK*; program(s) used to solve structure: *SHELXS86* (Sheldrick, 2008 ▶); program(s) used to refine structure: *SHELXL97* (Sheldrick, 2008 ▶); molecular graphics: *ORTEP-3 for Windows* (Farrugia, 2012 ▶); software used to prepare material for publication: *WinGX* (Farrugia, 2012 ▶).

## Supplementary Material

Crystal structure: contains datablock(s) I, global. DOI: 10.1107/S1600536813007307/xu5684sup1.cif

Structure factors: contains datablock(s) I. DOI: 10.1107/S1600536813007307/xu5684Isup2.hkl

Additional supplementary materials:  crystallographic information; 3D view; checkCIF report

## Figures and Tables

**Table 1:** Hydrogen-bond geometry (Å, °)

*D*—H⋯*A*	*D*—H	H⋯*A*	*D*⋯*A*	*D*—H⋯*A*
N1—H1*A*⋯O2	0.86	1.83	2.533 (4)	138
N2—H2⋯O3	0.86	1.82	2.530 (4)	138
N3—H3⋯O5	0.86	1.84	2.543 (4)	138
N4—H4*A*⋯O6	0.86	1.82	2.522 (4)	138
C20—H20⋯O2^i^	0.93	2.46	3.236 (5)	141
C46—H46⋯O3	0.93	2.37	3.085 (5)	134

Symmetry code: (i) 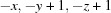.

**Fig. 1.**
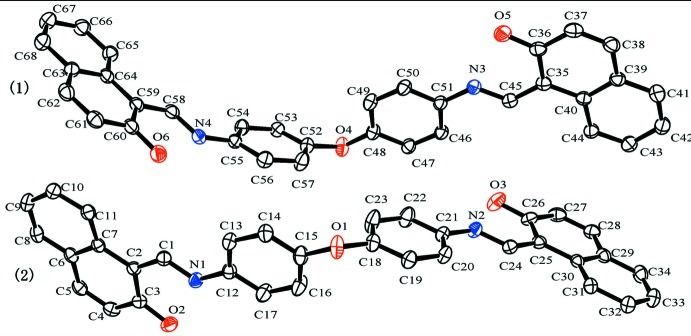
The title molecule with displacement ellipsoids for non–H atoms drawn at the 15% probability level.

**Fig. 2.**
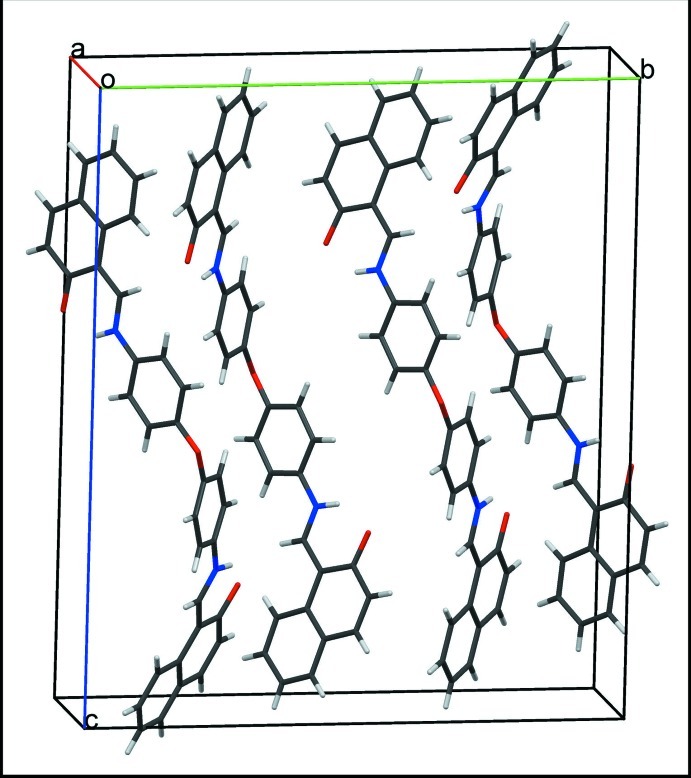
Packing of the molecules along the a–axis

## supplementary crystallographic information

### Comment

The most common method for preparation of Schiff base ligands is reacting stoichiometric amounts of a diamine and an aldehyde in various solvents. The reaction is carried out under stirring at reflux as described in the literature. These types of schiff bases with different coordinating sites may have wide application in the field of water treatment as they have a great capacity for complexation of transition metals (Izatt *et al.*, 1995, Kalcher *et al.*, 1995, Gilmartin *et al.*, 1995). They also serve as intermediates in certain enzymatic reactions and are also found in proteins that form the connective tissue (Khandar *et al.*, 2005, Chen *et al*., 2006) and in the pharmaceutical field (Souza *et al.*, 2005, Kidwai *et al.*, 2000). Their use as corrosion inhibitors (Ahamad *et al.*, 2010, Negm *et al.*, 2010, Zhenlan *et al.*, 2002) reveal their importance. Synthesized the compound,C34H24N2O3 is a condensation product of hydroxynaphtaldehyde with bifunctional aromatic diamine as shown in Fig (1). All the molecule are found in a single assymetric unit although, the oxygene atom is connecting the tow[(*E*)-(phenyliminio)methyl]naphthalen-2-olate units in (1) have the bond angle (C15—O1—C18) is equal to 116.3 (3)° and the dihedral angle of 75.5° between the planes defined as O(1)—C(18)—C(19)—C(20)—C(21)—C(22)—C(23) and O(1)—C(12)—C(13)—C(14)—C(15)—C(16)—C(17). In molecule (2) the bond angle (C15—O1—C18) is equal to 116.3 (3)° and the dihedral angle of 69.8° is found between the planes defined as O(4)—C(46)—C(47)—C(48)—C(49)—C(50)—C(51) and O(4)—C(52)—C(53)—C(54)—C(55)—C(56)—C(57). The bond angle between each imine phenyl plane and the attached hydroxynaphtaldehyde plane are 125.3 (3)° for C(1)—N(1)—C(12) and 126.0 (3)° for C(24)—N(2)—C(21). The bond lengths C(12)—N(1), C(1)—N(1), C(1)—C(2), C(15)—O(1).. and bond angles C(1)—N(1)—C(12), N(1)—C(1)—C(2), C(1)—C(2)—C(3), N(1)—C(12)—C(13) of one [(*E*)-(phenyliminio)methyl]naphthalen -2-olate moity are similar to the corresponding ones C(21)—N(2), C(24)—N(2), C(24)—C(25), C(18)—O(1) and C(24)—N(2)—C(21), N(2)—C(24)—C(25), C(24)—C(25)—C(26), N(2)—C(21)—C(22) of the second [(*E*)-(phenyliminio)methyl]naphthalen-2-olate. The bond distances shown in table 3 indicate that the C(1)—N(1) imine (C=N) bond length of 1.306 (4) Å agree with similar double bond usualy observed in related compounds (Girija *et al.*, 2004, Djamel *et al.*, 2011) but much shorter than single C—N 1.418 (4) Å of C(12)—N(1) (Gowda *et al.*, 2007) and for the molecule (2) the bond lengths C=N C(58)—N(4) is 1.295 (4) Å and bond single C(55)—N(4) is 1.410 (4) Å.

### Experimental

4,4'-Iminiomethylnaphthalen-2-olate[(*E*)-phenoxyphenyl] was prepared in proper literature (Issaadi *et al.*, 2005; Ghames *et al.*, 2006) by a condensation in ethanol (20 mL) of 2-hydroxy-1-naphthaldehyde (0.344 g, 2 mmol) with 4,4'-diaminodiphenyl ether (0.202 g, 1 mmol). The solution was stirred and refluxed for 4 h. The yellow precipitate was filtered, washed by a amount of ethanol and dried in vacuum. A single-crystal suitable for an X-ray structural analysis was obtained by slowly evaporation from dichloromethane-ethanol (1:1) solution at room temperature.

### Refinement

H atoms were included in geometric positions C—H = 0.93 Å and N—H = 0.86 Å, and refined by using a riding model with U_iso_(H) = 1.2_eq_(C,N).

### Crystal data

**Table:**

C_34_H_24_N_2_O_3_	*Z* = 4
*M**_r_* = 508.55	*F*(000) = 1064
Triclinic, *P*1	*D*_x_ = 1.332 Mg m^−^^3^
*a* = 5.292 (1) Å	Mo *K*α radiation, λ = 0.71073 Å
*b* = 20.203 (1) Å	Cell parameters from 8325 reflections
*c* = 23.863 (1) Å	θ = 1.0–25.4°
α = 87.853 (10)°	µ = 0.09 mm^−^^1^
β = 86.457 (10)°	*T* = 293 K
γ = 85.26 (1)°	Prism, yellow
*V* = 2536.4 (5) Å^3^	0.5 × 0.1 × 0.1 mm

### Data collection

**Table:**

Nonius KappaCCD diffractometer	4705 reflections with *I* > 2σ(*I*)
Radiation source: Enraf–Nonius FR590	*R*_int_ = 0.053
Graphite monochromator	θ_max_ = 25.3°, θ_min_ = 1.7°
Detector resolution: 9 pixels mm^-1^	*h* = −5→6
CCD rotation images, thick slices scans	*k* = −23→24
15547 measured reflections	*l* = −27→28
9159 independent reflections	

### Refinement

**Table:**

Refinement on *F*^2^	Primary atom site location: structure-invariant direct methods
Least-squares matrix: full	Secondary atom site location: difference Fourier map
*R*[*F*^2^ > 2σ(*F*^2^)] = 0.072	Hydrogen site location: inferred from neighbouring sites
*wR*(*F*^2^) = 0.218	H-atom parameters constrained
*S* = 1.02	*w* = 1/[σ^2^(*F*_o_^2^) + (0.1115*P*)^2^] where *P* = (*F*_o_^2^ + 2*F*_c_^2^)/3
9159 reflections	(Δ/σ)_max_ < 0.001
706 parameters	Δρ_max_ = 0.27 e Å^−^^3^
0 restraints	Δρ_min_ = −0.24 e Å^−^^3^

### Special details

**Table:**

Geometry. All e.s.d.'s (except the e.s.d. in the dihedral angle between two l.s. planes) are estimated using the full covariance matrix. The cell e.s.d.'s are taken into account individually in the estimation of e.s.d.'s in distances, angles and torsion angles; correlations between e.s.d.'s in cell parameters are only used when they are defined by crystal symmetry. An approximate (isotropic) treatment of cell e.s.d.'s is used for estimating e.s.d.'s involving l.s. planes.
Refinement. Refinement of *F*^2^ against ALL reflections. The weighted *R*-factor *wR* and goodness of fit *S* are based on *F*^2^, conventional *R*-factors *R* are based on *F*, with *F* set to zero for negative *F*^2^. The threshold expression of *F*^2^ > σ(*F*^2^) is used only for calculating *R*-factors(gt) *etc*. and is not relevant to the choice of reflections for refinement. *R*-factors based on *F*^2^ are statistically about twice as large as those based on *F*, and *R*- factors based on ALL data will be even larger.

### Fractional atomic coordinates and isotropic or equivalent isotropic displacement parameters (Å^2^)

**Table:**

	*x*	*y*	*z*	*U*_iso_*/*U*_eq_	
C1	0.1290 (6)	0.55264 (18)	0.24570 (14)	0.0560 (8)	
H1	0.0066	0.5853	0.2344	0.067*	
C2	0.3238 (5)	0.52973 (16)	0.20601 (13)	0.0517 (8)	
C3	0.5173 (6)	0.48181 (18)	0.22321 (15)	0.0584 (9)	
C4	0.7215 (6)	0.46177 (18)	0.18409 (17)	0.0650 (10)	
H4	0.8517	0.4315	0.1956	0.078*	
C5	0.7285 (6)	0.48602 (19)	0.13074 (16)	0.0648 (10)	
H5	0.8643	0.4719	0.1063	0.078*	
C6	0.5354 (6)	0.53258 (17)	0.11036 (14)	0.0563 (9)	
C7	0.3319 (5)	0.55482 (16)	0.14811 (13)	0.0506 (8)	
C8	0.5407 (7)	0.5556 (2)	0.05415 (15)	0.0681 (10)	
H8	0.6765	0.5413	0.0298	0.082*	
C9	0.3524 (7)	0.5983 (2)	0.03446 (15)	0.0694 (10)	
H9	0.3582	0.6124	−0.0031	0.083*	
C10	0.1510 (6)	0.62095 (19)	0.07083 (14)	0.0643 (9)	
H10	0.0226	0.6507	0.0577	0.077*	
C11	0.1418 (6)	0.59934 (18)	0.12612 (14)	0.0581 (9)	
H11	0.0051	0.6147	0.1498	0.07*	
C12	−0.0632 (6)	0.55183 (18)	0.34053 (14)	0.0571 (9)	
C13	−0.2802 (6)	0.59254 (19)	0.33162 (14)	0.0635 (9)	
H13	−0.3191	0.6059	0.2952	0.076*	
C14	−0.4405 (6)	0.6136 (2)	0.37675 (15)	0.0661 (10)	
H14	−0.586	0.6413	0.3706	0.079*	
C15	−0.3852 (6)	0.5937 (2)	0.43027 (15)	0.0659 (10)	
C16	−0.1728 (7)	0.5518 (2)	0.43944 (15)	0.0850 (13)	
H16	−0.1371	0.5376	0.4758	0.102*	
C17	−0.0130 (7)	0.5309 (2)	0.39481 (16)	0.0773 (12)	
H17	0.1304	0.5025	0.4012	0.093*	
C18	−0.4462 (6)	0.6394 (2)	0.52076 (14)	0.0619 (9)	
C19	−0.5430 (6)	0.6228 (2)	0.57295 (15)	0.0661 (10)	
H19	−0.6739	0.5946	0.5771	0.079*	
C20	−0.4471 (7)	0.6476 (2)	0.62007 (14)	0.0681 (10)	
H20	−0.5167	0.6369	0.6557	0.082*	
C21	−0.2479 (6)	0.68824 (18)	0.61417 (13)	0.0567 (9)	
C22	−0.1582 (7)	0.7059 (2)	0.56113 (16)	0.0826 (13)	
H22	−0.0294	0.7347	0.5563	0.099*	
C23	−0.2587 (8)	0.6812 (3)	0.51488 (16)	0.0941 (15)	
H23	−0.1963	0.6936	0.479	0.113*	
C24	−0.1706 (6)	0.69730 (17)	0.71287 (14)	0.0566 (8)	
H24	−0.2942	0.6683	0.7232	0.068*	
C25	−0.0345 (6)	0.72262 (17)	0.75547 (14)	0.0533 (8)	
C26	0.1549 (6)	0.76727 (18)	0.73981 (16)	0.0648 (10)	
C27	0.2941 (6)	0.79275 (19)	0.78256 (17)	0.0691 (10)	
H27	0.418	0.8218	0.7727	0.083*	
C28	0.2477 (6)	0.77516 (19)	0.83696 (17)	0.0667 (10)	
H28	0.3404	0.7929	0.8638	0.08*	
C29	0.0632 (6)	0.73074 (17)	0.85489 (14)	0.0570 (9)	
C30	−0.0812 (5)	0.70443 (16)	0.81407 (13)	0.0499 (8)	
C31	−0.2635 (6)	0.66099 (18)	0.83365 (14)	0.0586 (9)	
H31	−0.3635	0.6435	0.808	0.07*	
C32	−0.2978 (6)	0.6436 (2)	0.88968 (15)	0.0673 (10)	
H32	−0.4174	0.6139	0.9013	0.081*	
C33	−0.1559 (7)	0.6699 (2)	0.92902 (15)	0.0759 (11)	
H33	−0.182	0.6587	0.967	0.091*	
C34	0.0216 (7)	0.7123 (2)	0.91173 (15)	0.0725 (11)	
H34	0.1177	0.7295	0.9382	0.087*	
N1	0.1154 (5)	0.52958 (15)	0.29725 (11)	0.0598 (7)	
H1A	0.2254	0.4977	0.3059	0.072*	
N2	−0.1302 (5)	0.71287 (15)	0.65981 (12)	0.0633 (8)	
H2	−0.02	0.7412	0.6517	0.076*	
O1	−0.5535 (4)	0.61490 (15)	0.47444 (9)	0.0788 (8)	
O2	0.5137 (5)	0.45506 (13)	0.27369 (11)	0.0779 (8)	
O3	0.2058 (5)	0.78523 (16)	0.68792 (12)	0.0928 (9)	
C35	−0.4454 (6)	0.97036 (17)	0.69937 (14)	0.0551 (8)	
C36	−0.6474 (6)	1.01138 (18)	0.67549 (16)	0.0617 (9)	
C37	−0.8385 (6)	1.04351 (19)	0.71295 (18)	0.0698 (10)	
H37	−0.9717	1.0701	0.6982	0.084*	
C38	−0.8282 (6)	1.03579 (19)	0.76865 (17)	0.0696 (10)	
H38	−0.955	1.0575	0.7915	0.084*	
C39	−0.6313 (6)	0.99574 (18)	0.79435 (15)	0.0601 (9)	
C40	−0.4386 (6)	0.96220 (16)	0.75985 (14)	0.0544 (8)	
C41	−0.6260 (7)	0.9884 (2)	0.85341 (17)	0.0782 (11)	
H41	−0.7514	1.0112	0.8759	0.094*	
C42	−0.4421 (7)	0.9487 (2)	0.87806 (17)	0.0773 (11)	
H42	−0.4423	0.9441	0.917	0.093*	
C43	−0.2549 (7)	0.9155 (2)	0.84464 (16)	0.0718 (10)	
H43	−0.1292	0.8881	0.8614	0.086*	
C44	−0.2506 (6)	0.92227 (18)	0.78699 (15)	0.0613 (9)	
H44	−0.1202	0.8999	0.7656	0.074*	
C45	−0.2545 (6)	0.93931 (18)	0.66293 (15)	0.0589 (9)	
H45	−0.1244	0.9123	0.6785	0.071*	
C46	0.1470 (6)	0.87987 (19)	0.58422 (14)	0.0613 (9)	
H46	0.185	0.8755	0.6218	0.074*	
C47	0.3075 (6)	0.85007 (19)	0.54427 (14)	0.0622 (9)	
H47	0.4526	0.8247	0.5548	0.075*	
C48	0.2560 (6)	0.85735 (19)	0.48808 (14)	0.0612 (9)	
C49	0.0444 (7)	0.8959 (2)	0.47208 (14)	0.0689 (10)	
H49	0.0122	0.902	0.4343	0.083*	
C50	−0.1196 (6)	0.9252 (2)	0.51279 (14)	0.0647 (10)	
H50	−0.2635	0.9511	0.5022	0.078*	
C51	−0.0731 (6)	0.91681 (17)	0.56932 (13)	0.0558 (8)	
C52	0.3438 (6)	0.80954 (19)	0.39941 (14)	0.0603 (9)	
C53	0.4631 (6)	0.83115 (18)	0.35055 (15)	0.0614 (9)	
H53	0.6016	0.8565	0.3517	0.074*	
C54	0.3795 (6)	0.81564 (19)	0.29991 (15)	0.0634 (9)	
H54	0.4628	0.8301	0.2668	0.076*	
C55	0.1723 (6)	0.77871 (17)	0.29765 (14)	0.0563 (9)	
C56	0.0620 (7)	0.7547 (2)	0.34709 (16)	0.0701 (10)	
H56	−0.0708	0.7274	0.3463	0.084*	
C57	0.1468 (7)	0.7707 (2)	0.39782 (16)	0.0802 (12)	
H57	0.0691	0.7549	0.4311	0.096*	
C58	0.0878 (6)	0.79893 (18)	0.20050 (15)	0.0596 (9)	
H58	0.2089	0.8299	0.1969	0.072*	
C59	−0.0568 (6)	0.78884 (18)	0.15345 (14)	0.0570 (8)	
C60	−0.2439 (6)	0.74143 (19)	0.15925 (16)	0.0635 (9)	
C61	−0.3907 (6)	0.7320 (2)	0.11311 (17)	0.0702 (10)	
H61	−0.5122	0.7011	0.1166	0.084*	
C62	−0.3588 (7)	0.7667 (2)	0.06417 (17)	0.0723 (11)	
H62	−0.4582	0.759	0.0347	0.087*	
C63	−0.1774 (6)	0.81475 (19)	0.05649 (15)	0.0644 (10)	
C64	−0.0245 (6)	0.82639 (18)	0.10117 (14)	0.0579 (9)	
C65	0.1517 (6)	0.87515 (19)	0.09202 (16)	0.0674 (10)	
H65	0.2552	0.8838	0.1206	0.081*	
C66	0.1723 (7)	0.9098 (2)	0.04183 (18)	0.0778 (11)	
H66	0.2881	0.9421	0.037	0.093*	
C67	0.0239 (9)	0.8978 (2)	−0.00217 (18)	0.0876 (13)	
H67	0.0411	0.9213	−0.0363	0.105*	
C68	−0.1480 (8)	0.8509 (2)	0.00554 (17)	0.0831 (12)	
H68	−0.2479	0.8428	−0.0238	0.1*	
N3	−0.2527 (5)	0.94693 (14)	0.60848 (11)	0.0608 (7)	
H3	−0.373	0.9729	0.5951	0.073*	
N4	0.0568 (5)	0.76660 (15)	0.24782 (12)	0.0613 (7)	
H4A	−0.043	0.735	0.249	0.074*	
O4	0.4340 (4)	0.82707 (15)	0.44986 (10)	0.0769 (8)	
O5	−0.6624 (5)	1.02115 (13)	0.62214 (11)	0.0781 (8)	
O6	−0.2816 (5)	0.70620 (13)	0.20612 (11)	0.0746 (7)	

### Atomic displacement parameters (Å^2^)

**Table:**

	*U*^11^	*U*^22^	*U*^33^	*U*^12^	*U*^13^	*U*^23^
C1	0.0578 (18)	0.057 (2)	0.053 (2)	−0.0109 (15)	−0.0014 (15)	0.0001 (17)
C2	0.0541 (18)	0.047 (2)	0.053 (2)	−0.0073 (14)	0.0025 (14)	−0.0028 (16)
C3	0.062 (2)	0.052 (2)	0.060 (2)	−0.0048 (16)	−0.0047 (16)	−0.0001 (18)
C4	0.0572 (19)	0.053 (2)	0.083 (3)	−0.0002 (16)	0.0002 (17)	−0.005 (2)
C5	0.058 (2)	0.062 (2)	0.073 (3)	−0.0042 (17)	0.0120 (17)	−0.019 (2)
C6	0.0505 (18)	0.058 (2)	0.062 (2)	−0.0107 (15)	0.0028 (14)	−0.0129 (17)
C7	0.0493 (17)	0.050 (2)	0.053 (2)	−0.0116 (14)	0.0015 (13)	−0.0087 (16)
C8	0.067 (2)	0.084 (3)	0.054 (2)	−0.0145 (19)	0.0081 (16)	−0.020 (2)
C9	0.073 (2)	0.090 (3)	0.046 (2)	−0.016 (2)	−0.0022 (17)	−0.002 (2)
C10	0.068 (2)	0.067 (3)	0.058 (2)	−0.0054 (17)	−0.0045 (16)	−0.0005 (19)
C11	0.0567 (19)	0.061 (2)	0.056 (2)	−0.0021 (16)	0.0022 (15)	−0.0062 (17)
C12	0.0553 (19)	0.064 (2)	0.053 (2)	−0.0115 (16)	0.0006 (14)	−0.0047 (17)
C13	0.065 (2)	0.078 (3)	0.048 (2)	−0.0057 (18)	−0.0047 (15)	−0.0005 (18)
C14	0.060 (2)	0.081 (3)	0.057 (2)	−0.0014 (18)	−0.0025 (16)	−0.009 (2)
C15	0.0522 (19)	0.091 (3)	0.055 (2)	−0.0114 (18)	0.0009 (15)	−0.007 (2)
C16	0.077 (2)	0.128 (4)	0.047 (2)	0.003 (2)	0.0008 (18)	0.014 (2)
C17	0.072 (2)	0.098 (3)	0.057 (2)	0.010 (2)	−0.0005 (18)	0.010 (2)
C18	0.0525 (18)	0.082 (3)	0.051 (2)	−0.0034 (17)	−0.0023 (15)	−0.0022 (19)
C19	0.064 (2)	0.080 (3)	0.055 (2)	−0.0135 (18)	0.0008 (16)	0.004 (2)
C20	0.078 (2)	0.083 (3)	0.043 (2)	−0.012 (2)	−0.0002 (16)	0.0086 (19)
C21	0.0595 (19)	0.065 (2)	0.045 (2)	−0.0040 (16)	−0.0066 (14)	0.0058 (17)
C22	0.084 (3)	0.112 (4)	0.058 (2)	−0.045 (2)	−0.0023 (19)	0.006 (2)
C23	0.101 (3)	0.138 (4)	0.048 (2)	−0.052 (3)	0.003 (2)	0.007 (3)
C24	0.0595 (18)	0.055 (2)	0.054 (2)	−0.0006 (15)	−0.0057 (15)	0.0045 (17)
C25	0.0551 (18)	0.048 (2)	0.056 (2)	0.0008 (15)	−0.0085 (14)	0.0019 (16)
C26	0.069 (2)	0.056 (2)	0.069 (3)	−0.0031 (17)	−0.0140 (18)	0.015 (2)
C27	0.066 (2)	0.056 (2)	0.088 (3)	−0.0129 (17)	−0.0173 (19)	0.003 (2)
C28	0.064 (2)	0.062 (3)	0.076 (3)	−0.0021 (17)	−0.0198 (18)	−0.009 (2)
C29	0.0553 (18)	0.056 (2)	0.060 (2)	0.0042 (15)	−0.0092 (15)	−0.0086 (17)
C30	0.0521 (17)	0.047 (2)	0.0496 (19)	0.0066 (14)	−0.0082 (13)	−0.0065 (15)
C31	0.0610 (19)	0.064 (2)	0.051 (2)	−0.0047 (16)	−0.0044 (15)	−0.0069 (17)
C32	0.070 (2)	0.078 (3)	0.054 (2)	−0.0095 (19)	0.0011 (16)	−0.002 (2)
C33	0.082 (3)	0.099 (3)	0.047 (2)	−0.007 (2)	−0.0067 (18)	−0.003 (2)
C34	0.079 (2)	0.084 (3)	0.055 (2)	0.000 (2)	−0.0176 (18)	−0.015 (2)
N1	0.0627 (16)	0.0613 (19)	0.0543 (18)	−0.0053 (13)	0.0028 (13)	0.0029 (15)
N2	0.0693 (17)	0.069 (2)	0.0527 (18)	−0.0124 (15)	−0.0096 (13)	0.0084 (16)
O1	0.0572 (14)	0.133 (3)	0.0487 (15)	−0.0162 (14)	0.0013 (10)	−0.0195 (15)
O2	0.0898 (17)	0.0694 (18)	0.0713 (18)	0.0043 (14)	−0.0028 (13)	0.0123 (14)
O3	0.103 (2)	0.103 (2)	0.076 (2)	−0.0363 (17)	−0.0163 (15)	0.0316 (18)
C35	0.0551 (18)	0.051 (2)	0.061 (2)	−0.0092 (15)	−0.0077 (15)	−0.0036 (17)
C36	0.065 (2)	0.049 (2)	0.073 (3)	−0.0085 (16)	−0.0139 (17)	0.0024 (19)
C37	0.060 (2)	0.057 (2)	0.090 (3)	0.0042 (17)	−0.0059 (18)	0.007 (2)
C38	0.067 (2)	0.061 (3)	0.077 (3)	0.0043 (18)	0.0081 (18)	−0.002 (2)
C39	0.0588 (19)	0.053 (2)	0.067 (2)	−0.0041 (16)	0.0017 (16)	0.0013 (18)
C40	0.0531 (18)	0.047 (2)	0.064 (2)	−0.0082 (15)	−0.0041 (15)	−0.0014 (17)
C41	0.081 (3)	0.080 (3)	0.071 (3)	0.001 (2)	0.011 (2)	−0.008 (2)
C42	0.083 (3)	0.087 (3)	0.060 (2)	−0.002 (2)	0.0001 (19)	0.001 (2)
C43	0.075 (2)	0.074 (3)	0.066 (3)	0.0015 (19)	−0.0118 (18)	0.000 (2)
C44	0.0585 (19)	0.065 (2)	0.060 (2)	0.0009 (16)	−0.0050 (15)	−0.0002 (18)
C45	0.0586 (19)	0.057 (2)	0.061 (2)	−0.0058 (16)	−0.0084 (15)	0.0023 (18)
C46	0.070 (2)	0.065 (2)	0.050 (2)	−0.0059 (18)	−0.0109 (16)	0.0011 (18)
C47	0.064 (2)	0.069 (3)	0.055 (2)	−0.0068 (17)	−0.0120 (16)	−0.0008 (19)
C48	0.0580 (19)	0.075 (3)	0.051 (2)	−0.0110 (17)	−0.0068 (15)	0.0009 (18)
C49	0.072 (2)	0.086 (3)	0.049 (2)	−0.007 (2)	−0.0108 (16)	0.004 (2)
C50	0.066 (2)	0.074 (3)	0.054 (2)	−0.0015 (18)	−0.0115 (16)	0.0052 (19)
C51	0.0633 (19)	0.056 (2)	0.049 (2)	−0.0071 (16)	−0.0082 (15)	−0.0008 (17)
C52	0.0552 (19)	0.074 (3)	0.051 (2)	−0.0032 (17)	−0.0025 (15)	−0.0034 (18)
C53	0.0597 (19)	0.063 (2)	0.062 (2)	−0.0072 (16)	−0.0063 (16)	−0.0010 (19)
C54	0.060 (2)	0.074 (3)	0.056 (2)	−0.0096 (18)	0.0038 (15)	−0.0034 (19)
C55	0.0566 (18)	0.057 (2)	0.054 (2)	0.0053 (16)	−0.0018 (15)	−0.0051 (17)
C56	0.071 (2)	0.072 (3)	0.070 (3)	−0.0206 (19)	−0.0028 (18)	0.001 (2)
C57	0.090 (3)	0.101 (3)	0.052 (2)	−0.033 (2)	−0.0014 (19)	0.008 (2)
C58	0.0624 (19)	0.052 (2)	0.063 (2)	0.0050 (16)	0.0003 (16)	−0.0077 (18)
C59	0.0593 (19)	0.055 (2)	0.056 (2)	0.0047 (16)	−0.0038 (15)	−0.0116 (17)
C60	0.069 (2)	0.055 (2)	0.066 (2)	0.0023 (17)	−0.0001 (17)	−0.0170 (19)
C61	0.066 (2)	0.068 (3)	0.078 (3)	−0.0081 (18)	−0.0080 (19)	−0.015 (2)
C62	0.068 (2)	0.078 (3)	0.071 (3)	0.004 (2)	−0.0161 (18)	−0.020 (2)
C63	0.068 (2)	0.066 (3)	0.058 (2)	0.0126 (18)	−0.0084 (16)	−0.0061 (19)
C64	0.0593 (19)	0.056 (2)	0.057 (2)	0.0076 (16)	0.0005 (15)	−0.0121 (17)
C65	0.068 (2)	0.069 (3)	0.065 (2)	−0.0031 (18)	−0.0019 (17)	−0.004 (2)
C66	0.086 (3)	0.070 (3)	0.076 (3)	−0.006 (2)	0.006 (2)	0.000 (2)
C67	0.107 (3)	0.090 (4)	0.065 (3)	−0.001 (3)	−0.006 (2)	0.011 (2)
C68	0.095 (3)	0.087 (3)	0.067 (3)	0.011 (2)	−0.022 (2)	−0.007 (2)
N3	0.0677 (17)	0.060 (2)	0.0553 (19)	0.0005 (14)	−0.0124 (13)	−0.0005 (15)
N4	0.0678 (17)	0.0550 (19)	0.0613 (19)	−0.0043 (14)	−0.0017 (14)	−0.0100 (15)
O4	0.0625 (14)	0.113 (2)	0.0553 (16)	−0.0029 (14)	−0.0054 (11)	−0.0138 (15)
O5	0.0899 (17)	0.0749 (19)	0.0697 (18)	0.0072 (14)	−0.0274 (13)	0.0003 (14)
O6	0.0875 (17)	0.0694 (18)	0.0671 (17)	−0.0133 (13)	0.0068 (13)	−0.0058 (14)

### Geometric parameters (Å, º)

**Table:**

C1—N1	1.299 (4)	C35—C45	1.414 (5)
C1—C2	1.417 (4)	C35—C36	1.431 (5)
C1—H1	0.93	C35—C40	1.449 (4)
C2—C3	1.419 (4)	C36—O5	1.287 (4)
C2—C7	1.453 (4)	C36—C37	1.438 (5)
C3—O2	1.301 (4)	C37—C38	1.336 (5)
C3—C4	1.426 (5)	C37—H37	0.93
C4—C5	1.346 (5)	C38—C39	1.421 (5)
C4—H4	0.93	C38—H38	0.93
C5—C6	1.426 (5)	C39—C41	1.413 (5)
C5—H5	0.93	C39—C40	1.415 (5)
C6—C8	1.403 (5)	C40—C44	1.403 (4)
C6—C7	1.415 (4)	C41—C42	1.357 (5)
C7—C11	1.406 (4)	C41—H41	0.93
C8—C9	1.358 (5)	C42—C43	1.378 (5)
C8—H8	0.93	C42—H42	0.93
C9—C10	1.391 (5)	C43—C44	1.376 (5)
C9—H9	0.93	C43—H43	0.93
C10—C11	1.374 (4)	C44—H44	0.93
C10—H10	0.93	C45—N3	1.302 (4)
C11—H11	0.93	C45—H45	0.93
C12—C13	1.379 (5)	C46—C47	1.360 (5)
C12—C17	1.382 (5)	C46—C51	1.388 (5)
C12—N1	1.416 (4)	C46—H46	0.93
C13—C14	1.386 (5)	C47—C48	1.385 (4)
C13—H13	0.93	C47—H47	0.93
C14—C15	1.366 (5)	C48—C49	1.375 (5)
C14—H14	0.93	C48—O4	1.391 (4)
C15—C16	1.373 (5)	C49—C50	1.379 (5)
C15—O1	1.394 (4)	C49—H49	0.93
C16—C17	1.375 (5)	C50—C51	1.388 (4)
C16—H16	0.93	C50—H50	0.93
C17—H17	0.93	C51—N3	1.409 (4)
C18—C23	1.353 (5)	C52—C57	1.359 (5)
C18—C19	1.358 (4)	C52—C53	1.366 (5)
C18—O1	1.398 (4)	C52—O4	1.390 (4)
C19—C20	1.389 (5)	C53—C54	1.368 (5)
C19—H19	0.93	C53—H53	0.93
C20—C21	1.386 (5)	C54—C55	1.381 (5)
C20—H20	0.93	C54—H54	0.93
C21—C22	1.369 (5)	C55—C56	1.374 (5)
C21—N2	1.413 (4)	C55—N4	1.409 (4)
C22—C23	1.381 (5)	C56—C57	1.375 (5)
C22—H22	0.93	C56—H56	0.93
C23—H23	0.93	C57—H57	0.93
C24—N2	1.302 (4)	C58—N4	1.292 (4)
C24—C25	1.416 (5)	C58—C59	1.427 (5)
C24—H24	0.93	C58—H58	0.93
C25—C26	1.427 (5)	C59—C60	1.430 (5)
C25—C30	1.444 (4)	C59—C64	1.444 (5)
C26—O3	1.296 (4)	C60—O6	1.317 (4)
C26—C27	1.429 (5)	C60—C61	1.413 (5)
C27—C28	1.346 (5)	C61—C62	1.348 (5)
C27—H27	0.93	C61—H61	0.93
C28—C29	1.416 (5)	C62—C63	1.420 (5)
C28—H28	0.93	C62—H62	0.93
C29—C34	1.402 (5)	C63—C68	1.402 (5)
C29—C30	1.419 (4)	C63—C64	1.416 (5)
C30—C31	1.405 (4)	C64—C65	1.414 (5)
C31—C32	1.375 (4)	C65—C66	1.367 (5)
C31—H31	0.93	C65—H65	0.93
C32—C33	1.385 (5)	C66—C67	1.389 (6)
C32—H32	0.93	C66—H66	0.93
C33—C34	1.358 (5)	C67—C68	1.367 (6)
C33—H33	0.93	C67—H67	0.93
C34—H34	0.93	C68—H68	0.93
N1—H1A	0.86	N3—H3	0.86
N2—H2	0.86	N4—H4A	0.86
			
N1—C1—C2	122.3 (3)	C45—C35—C36	118.7 (3)
N1—C1—H1	118.9	C45—C35—C40	121.5 (3)
C2—C1—H1	118.9	C36—C35—C40	119.8 (3)
C1—C2—C3	119.4 (3)	O5—C36—C35	122.7 (3)
C1—C2—C7	121.5 (3)	O5—C36—C37	119.1 (3)
C3—C2—C7	119.1 (3)	C35—C36—C37	118.2 (3)
O2—C3—C2	122.0 (3)	C38—C37—C36	121.2 (3)
O2—C3—C4	118.7 (3)	C38—C37—H37	119.4
C2—C3—C4	119.3 (3)	C36—C37—H37	119.4
C5—C4—C3	121.0 (3)	C37—C38—C39	122.7 (3)
C5—C4—H4	119.5	C37—C38—H38	118.7
C3—C4—H4	119.5	C39—C38—H38	118.7
C4—C5—C6	122.4 (3)	C41—C39—C40	119.6 (3)
C4—C5—H5	118.8	C41—C39—C38	121.4 (3)
C6—C5—H5	118.8	C40—C39—C38	119.0 (3)
C8—C6—C7	119.8 (3)	C44—C40—C39	117.1 (3)
C8—C6—C5	121.7 (3)	C44—C40—C35	123.8 (3)
C7—C6—C5	118.5 (3)	C39—C40—C35	119.1 (3)
C11—C7—C6	116.8 (3)	C42—C41—C39	121.5 (4)
C11—C7—C2	123.5 (3)	C42—C41—H41	119.2
C6—C7—C2	119.7 (3)	C39—C41—H41	119.2
C9—C8—C6	121.6 (3)	C41—C42—C43	119.1 (4)
C9—C8—H8	119.2	C41—C42—H42	120.4
C6—C8—H8	119.2	C43—C42—H42	120.4
C8—C9—C10	119.6 (3)	C44—C43—C42	121.2 (4)
C8—C9—H9	120.2	C44—C43—H43	119.4
C10—C9—H9	120.2	C42—C43—H43	119.4
C11—C10—C9	119.9 (3)	C43—C44—C40	121.5 (3)
C11—C10—H10	120	C43—C44—H44	119.3
C9—C10—H10	120	C40—C44—H44	119.3
C10—C11—C7	122.3 (3)	N3—C45—C35	122.9 (3)
C10—C11—H11	118.9	N3—C45—H45	118.6
C7—C11—H11	118.9	C35—C45—H45	118.6
C13—C12—C17	118.9 (3)	C47—C46—C51	120.5 (3)
C13—C12—N1	124.1 (3)	C47—C46—H46	119.8
C17—C12—N1	116.9 (3)	C51—C46—H46	119.8
C12—C13—C14	120.2 (3)	C46—C47—C48	120.4 (3)
C12—C13—H13	119.9	C46—C47—H47	119.8
C14—C13—H13	119.9	C48—C47—H47	119.8
C15—C14—C13	120.1 (3)	C49—C48—C47	120.2 (3)
C15—C14—H14	119.9	C49—C48—O4	123.0 (3)
C13—C14—H14	119.9	C47—C48—O4	116.7 (3)
C14—C15—C16	120.0 (3)	C48—C49—C50	119.2 (3)
C14—C15—O1	118.4 (3)	C48—C49—H49	120.4
C16—C15—O1	121.5 (3)	C50—C49—H49	120.4
C15—C16—C17	120.0 (3)	C49—C50—C51	121.0 (3)
C15—C16—H16	120	C49—C50—H50	119.5
C17—C16—H16	120	C51—C50—H50	119.5
C16—C17—C12	120.6 (3)	C50—C51—C46	118.7 (3)
C16—C17—H17	119.7	C50—C51—N3	117.7 (3)
C12—C17—H17	119.7	C46—C51—N3	123.6 (3)
C23—C18—C19	119.6 (3)	C57—C52—C53	120.0 (3)
C23—C18—O1	122.0 (3)	C57—C52—O4	121.9 (3)
C19—C18—O1	118.3 (3)	C53—C52—O4	118.1 (3)
C18—C19—C20	120.2 (3)	C52—C53—C54	120.2 (3)
C18—C19—H19	119.9	C52—C53—H53	119.9
C20—C19—H19	119.9	C54—C53—H53	119.9
C21—C20—C19	120.2 (3)	C53—C54—C55	120.4 (3)
C21—C20—H20	119.9	C53—C54—H54	119.8
C19—C20—H20	119.9	C55—C54—H54	119.8
C22—C21—C20	118.6 (3)	C56—C55—C54	118.6 (3)
C22—C21—N2	117.5 (3)	C56—C55—N4	117.1 (3)
C20—C21—N2	123.9 (3)	C54—C55—N4	124.2 (3)
C21—C22—C23	120.1 (3)	C55—C56—C57	120.4 (3)
C21—C22—H22	119.9	C55—C56—H56	119.8
C23—C22—H22	119.9	C57—C56—H56	119.8
C18—C23—C22	121.2 (3)	C52—C57—C56	120.2 (3)
C18—C23—H23	119.4	C52—C57—H57	119.9
C22—C23—H23	119.4	C56—C57—H57	119.9
N2—C24—C25	122.8 (3)	N4—C58—C59	122.4 (3)
N2—C24—H24	118.6	N4—C58—H58	118.8
C25—C24—H24	118.6	C59—C58—H58	118.8
C24—C25—C26	118.8 (3)	C58—C59—C60	118.6 (3)
C24—C25—C30	122.0 (3)	C58—C59—C64	121.9 (3)
C26—C25—C30	119.2 (3)	C60—C59—C64	119.5 (3)
O3—C26—C25	122.0 (3)	O6—C60—C61	119.3 (3)
O3—C26—C27	118.9 (3)	O6—C60—C59	121.9 (3)
C25—C26—C27	119.1 (3)	C61—C60—C59	118.8 (4)
C28—C27—C26	120.7 (3)	C62—C61—C60	121.6 (4)
C28—C27—H27	119.6	C62—C61—H61	119.2
C26—C27—H27	119.6	C60—C61—H61	119.2
C27—C28—C29	122.6 (3)	C61—C62—C63	121.7 (3)
C27—C28—H28	118.7	C61—C62—H62	119.1
C29—C28—H28	118.7	C63—C62—H62	119.1
C34—C29—C28	121.5 (3)	C68—C63—C64	119.5 (4)
C34—C29—C30	119.7 (3)	C68—C63—C62	121.1 (4)
C28—C29—C30	118.8 (3)	C64—C63—C62	119.3 (3)
C31—C30—C29	117.0 (3)	C65—C64—C63	117.6 (3)
C31—C30—C25	123.5 (3)	C65—C64—C59	123.5 (3)
C29—C30—C25	119.5 (3)	C63—C64—C59	119.0 (3)
C32—C31—C30	121.7 (3)	C66—C65—C64	121.0 (4)
C32—C31—H31	119.2	C66—C65—H65	119.5
C30—C31—H31	119.2	C64—C65—H65	119.5
C31—C32—C33	120.5 (4)	C65—C66—C67	121.4 (4)
C31—C32—H32	119.7	C65—C66—H66	119.3
C33—C32—H32	119.7	C67—C66—H66	119.3
C34—C33—C32	119.5 (3)	C68—C67—C66	118.9 (4)
C34—C33—H33	120.3	C68—C67—H67	120.6
C32—C33—H33	120.3	C66—C67—H67	120.6
C33—C34—C29	121.6 (3)	C67—C68—C63	121.7 (4)
C33—C34—H34	119.2	C67—C68—H68	119.2
C29—C34—H34	119.2	C63—C68—H68	119.2
C1—N1—C12	126.3 (3)	C45—N3—C51	126.4 (3)
C1—N1—H1A	116.9	C45—N3—H3	116.8
C12—N1—H1A	116.9	C51—N3—H3	116.8
C24—N2—C21	127.1 (3)	C58—N4—C55	125.6 (3)
C24—N2—H2	116.5	C58—N4—H4A	117.2
C21—N2—H2	116.5	C55—N4—H4A	117.2
C15—O1—C18	116.5 (2)	C52—O4—C48	116.3 (3)

### Hydrogen-bond geometry (Å, º)

**Table:**

*D*—H···*A*	*D*—H	H···*A*	*D*···*A*	*D*—H···*A*
N1—H1*A*···O2	0.86	1.83	2.533 (4)	138
N2—H2···O3	0.86	1.82	2.530 (4)	138
N3—H3···O5	0.86	1.84	2.543 (4)	138
N4—H4*A*···O6	0.86	1.82	2.522 (4)	138
C20—H20···O2^i^	0.93	2.46	3.236 (5)	141
C46—H46···O3	0.93	2.37	3.085 (5)	134

Symmetry code: (i) −*x*, −*y*+1, −*z*+1.
